# Case Report: Intellectual disability and borderline intellectual functioning in two sisters with a 12p11.22 loss

**DOI:** 10.3389/fgene.2024.1355823

**Published:** 2024-04-02

**Authors:** Haemi Choi, Jeong-A. Kim, Kyung-Ok Cho, Hyun Jung Kim, Min-Hyeon Park

**Affiliations:** ^1^ Department of Psychiatry, Eunpyeong St. Mary’s Hospital, College of Medicine, The Catholic University of Korea, Seoul, Republic of Korea; ^2^ Department of Pharmacology, College of Medicine, The Catholic University of Korea, Seoul, Republic of Korea; ^3^ Department of Biomedicine & Health Sciences, The Catholic University of Korea, Seoul, Republic of Korea; ^4^ Catholic Neuroscience Institute, The Catholic University of Korea, Seoul, Republic of Korea; ^5^ Institute for Aging and Metabolic Diseases, The Catholic University of Korea, Seoul, Republic of Korea; ^6^ Department of Psychiatry, Harvard Medical School, Boston, MA, United States; ^7^ Division of Psychotic Disorders, McLean Hospital, Belmont, MA, United States

**Keywords:** cognitive impairment, neurodevelopmental disorder, 12p deletion, CNV, chromosomal microarray analysis

## Abstract

Multiple genome sequencing studies have identified genetic abnormalities as major causes of severe intellectual disability (ID). However, many children affected by mild ID and borderline intellectual functioning (BIF) lack a genetic diagnosis because known causative ID genetic mutations have not been identified or the role of genetic variants in mild cases is less understood. Genetic variant testing in mild cases is necessary to provide information on prognosis and risk of occurrence. In this study, we report two sibling patients who were 5 years 9 months old and 3 years 3 months old and presented to the hospital due to developmental delay. Clinical assessment and chromosomal microarray analysis were performed. The patients were diagnosed with mild intellectual disability (ID) and borderline intellectual functioning (BIF). Genetic analysis identified a loss of 12p11.22, including the *OVCH1-AS1*, *OVCH1*, and *TMTC1* genes, which was the only variant that occurred in both sisters. Identical variants were found in their father with probable BIF. Neither patient presented any brain structural abnormalities or dysmorphism, and no exogenous factors or parenting problems were reported. Thus, loss of 12p11.22 may be associated with our patients’ cognitive impairment. The *OVCH1*, *OVCH1-AS1* and *TMTC1* variants identified in this study are the most likely disease-causing genes in the sisters. Our findings may expand as yet limited knowledge on mild ID and BIF causative variants, which would further support the diagnosis even if the severity is mild.

## 1 Introduction

Intellectual disability (ID) is characterized by significant impairments in both intellectual and adaptive functioning, which refers to the ability to learn, reason, and solve problems, and functioning in conceptual, social, and practical domains (American Psychiatric Association, 2013). According to intelligence quotient (IQ) scores, severity is classified into mild (IQ 50-69), moderate (IQ 35-49), severe (IQ 20-34) and profound (IQ 0-19). Between 1% and 3% of the global population is affected by this type of developmental disorder (Leonard and Wen, 2002; Maulik et al., 2011); of all cases, approximately 85% are classified as having mild severity (Bhasin et al., 2006). Furthermore, it commonly affects 3.2% of children and adolescents (Olusanya et al., 2020), and 30%–50% of children and adolescents have a relative risk of comorbid mental disorder associated with ID (Einfeld et al., 2011).

Individuals with an IQ of 70–85 (between ID and normal intellectual functioning) are classified as borderline intellectual functioning (BIF) (Wieland and Zitman, 2016). However, children with BIF face difficulties in school accomplishments and social participation due to limitations in learning, social skills, and emotional competencies (Baglio et al., 2016; Contena and Taddei, 2017; Predescu et al., 2020). Therefore, they run a significant risk of dropping out of school and have a higher chance of having psychiatric problems in adulthood (Douma et al., 2007; Fernell and Ek, 2010; Gigi et al., 2014; Hassiotis et al., 2019). BIF is a prevalent condition that affects approximately 12%–14% of the population (Fernell and Gillberg, 2020). Nevertheless, children with mild ID and BIF may not be recognized until they are in school and have difficulty with academics.

To date, the underlying causes of these conditions are still unclear, although the prevailing hypothesis for the etiology of ID is complex interactions between multiple genetic and environmental factors that interfere with typical brain development; for example, different kinds of variations include mutations, deletions, and copy number variants, and exogenous factors include maternal infections, delivery complications, and exposure to drugs or alcohol while pregnant (Faustman et al., 2000; van Loo and Martens, 2007; De Felice et al., 2015).

One of the strategies utilized to unravel the bases of ID is chromosomal microarrays. Chromosomal microarray analysis (CMA) is a molecular genetic tool that can identify gains and losses in the number of microscopic DNA replications that would otherwise be detected by chromosomal karyotype analysis (Batzir et al., 2015). Changes in the number of microscopic DNA replications, copy number variants (CNVs), are linked to human diseases and health (Harel and Lupski, 2018; Hu et al., 2018). CNVs are classified into common and rare CNVs according to the population frequency. A growing body of evidence suggests that rare CNVs significantly contribute to the genetic basis of ID (Di Gregorio et al., 2017; Kessi et al., 2018). Identifying disease-associated CNVs can help further elucidate the pathophysiological mechanisms of ID. Hence, CMA has been widely utilized as the initial diagnostic process for patients with ID, as it considerably improves the diagnostic yield for children affected with ID (Manning et al., 2010; Miller et al., 2010; Kearney et al., 2011; Michelson et al., 2011).

Multiple genome sequencing studies have identified that the main causes of severe ID are genetic abnormalities, with reported diagnostic results ranging from 13% to 42% in predominantly severe ID (Deciphering Developmental Disorders, 2017). For mild ID and BIF, however, the role of genetic variants is less studied but is predicted to be less important (Vissers et al., 2016). A population-based study reported that chromosomal abnormalities account for approximately 40% of severe ID and 10% of mild ID cases (Karam et al., 2015). In another population-based study, the likely causative variants in known ID genes were significantly more common in more severe ID cases than in mild ID cases (Kurki et al., 2019). The authors suggested that either the etiology of mild ID is more complex or the genetic variants that cause mild ID differ slightly from those that cause more severe ID (Kurki et al., 2019). Many children affected by mild ID or BIF lack a genetic diagnosis because known causative ID genes were not identified or the role of genetic variants in mild cases is less understood. Therefore, CNV testing in mild ID and BIF is necessary to provide information on prognosis and risk of occurrence as well as to prevent unnecessary intrusive testing.

Here, we describe two sisters with mild ID and BIF. Loss of the 12p11.22 region, including the *OVCH1-AS1*, *OVCH1*, and *TMTC1* genes, was identified in both patients. Furthermore, identical CNVs were found in their father, who had probable BIF. This case highlights the importance of identifying the underlying genetic causes of mild ID and BIF.

## 2 Case description

### 2.1 Patient 1

**FIGURE 1:**
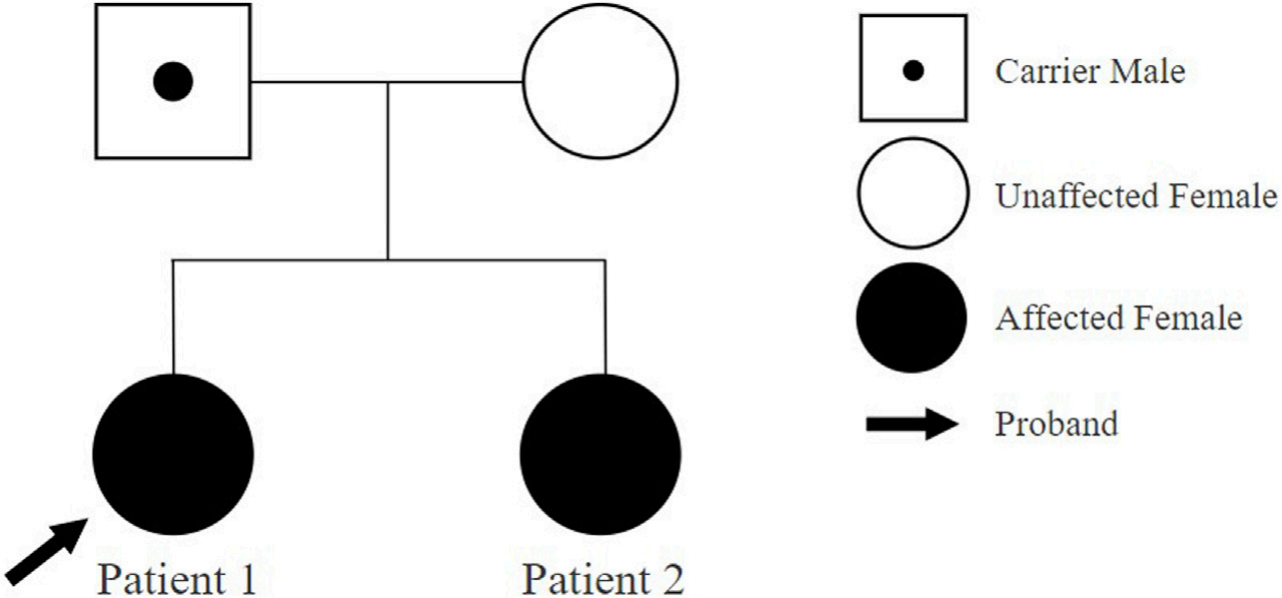
Family pedigree of patients.

**FIGURE 2:**
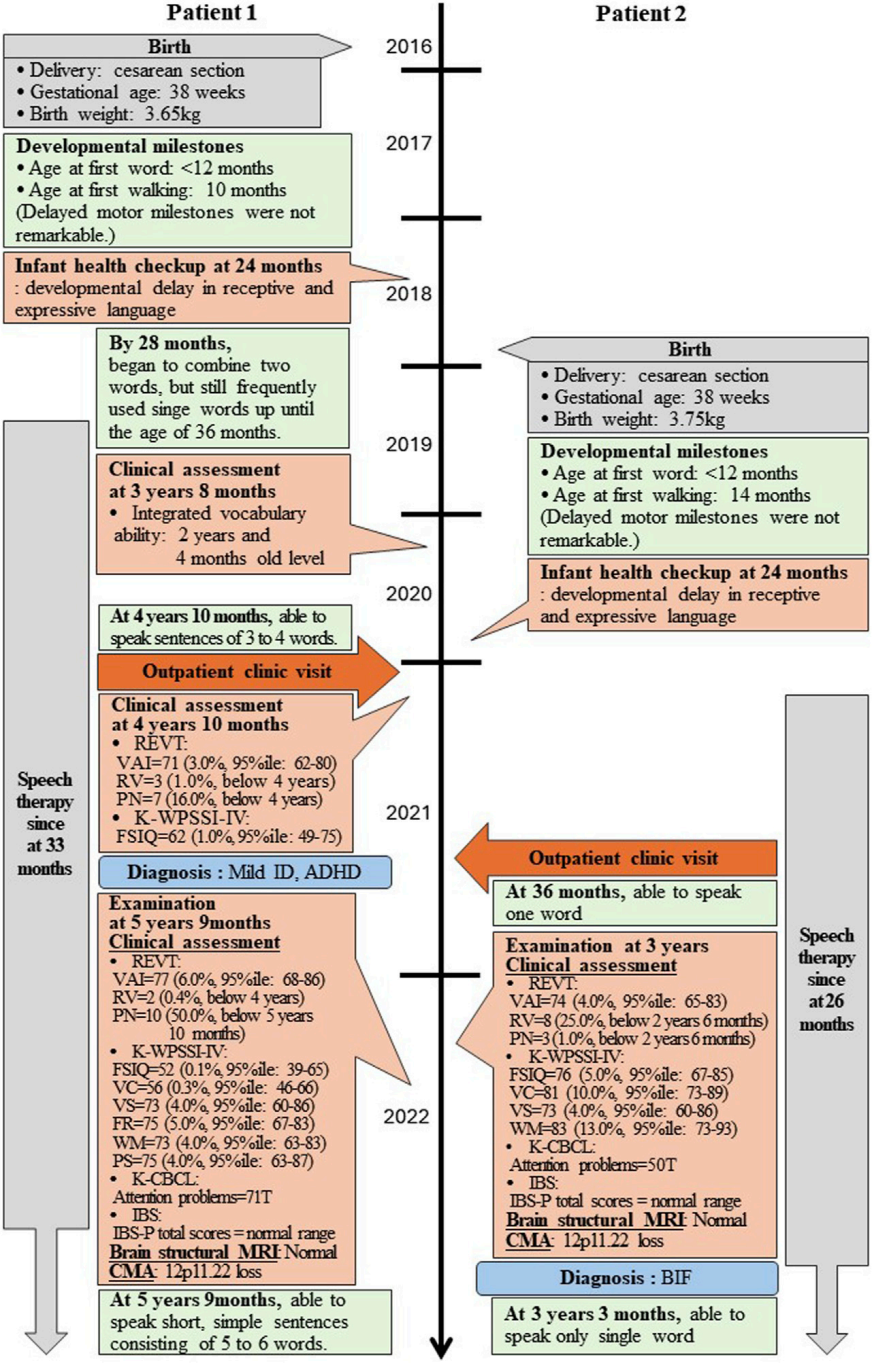
Timeline of two patients’ assessment and outcomes. *REVT*: receptive and expressive vocabulary test; *VAI*: vocabulary ability index; *RV*: receptive vocabulary; *PN*: picture naming; *K-WPSSI-IV*: Korean Wechsler preschool and primary scale of intelligence fourth edition; *FSIQ*: full scale intelligence quotient; *VC*: vocabulary comprehensive; VS: visual spatial; *FR*: fluid reasoning; *WM*: working memory; *PS*: processing speed; *CBCL*: child behavior checklist; *IBS*: interactive behavioral scale; *MRI*: magnetic resonance imaging; *CMA*: chromosomal microarray analysis; *ID*: intellectual disability; *BIF*: borderline intellectual functioning; *ADHD*: attention deficit hyperactivity disorder.

Patient 1 is a 5-year-and 9-month-old female, who initially presented to the outpatient clinic of the Catholic University of Korea, Eunpyeong St. Mary’s Hospital at 4 years and 10 months, due to developmental delays. She was the first child born to healthy nonconsanguineous Korean parents (Figure 1). The mother’s prenatal history was unremarkable with no alcohol, smoking, or drug use during pregnancy. Patient 1 was delivered at 38 weeks of gestation via cesarean section, with a birth weight of 3.65 kg. A timeline of assessment and outcomes is outlined in Figure 2.

She achieved early language milestones, speaking her first word before 12 months and walking independently by 10 months, with no significant delays in motor development or seizure occurrences. However, her mother reported concerns about her language development compared to peers at 20 months. By the 24-month infant health checkup, pronounced developmental delays were noted in both receptive and expressive language; she began to form two-word phrases at 28 months but continued to use single words frequently until 36 months.

Intervention with speech therapy began at 33 months. At 3 years and 8 months, she was assessed to have an integrated vocabulary ability delayed by 1 year and 6 months. By 4 years and 10 months, symptoms of inattention and hyperactivity were observed, intensifying concerns regarding her developmental progression. A clinical psychological evaluation confirmed developmental delays in intelligence and language, with a diagnosis of mild ID characterized by a full-scale intelligence quotient (FSIQ) of 62. She also presented with attention deficit hyperactivity disorder (ADHD). At this time, her language abilities remained below the 4-year-old standard; she could form sentences of 3–4 words.

At 5 years and 9 months, she has undergone comprehensive investigation, including clinical assessment, magnetic resonance imaging (MRI), and CMA. The clinical results revealed scores of a FSIQ of 52, along with sub-scores of vocabulary comprehension (VC) at 56, visual spatial (VS) at 73, fluid reasoning (FR) at 75, working memory (WM) at 73, and processing speed (PS) at 75. Despite ongoing speech therapy initiated at 33 months, her receptive language skills still remained below 4-year-old level, while expressive skills had improved to the level of a 5-year-and 10-month-old. To date, she has been able to speak short, simple sentences consisting of 5–6 words.

The interactive behavioral scale (IBS), completed by her mother, indicated normal parental and child interactive behaviors. The family was described as having a close and supportive relationship. Clinically, she presented with no dysmorphic features, and brain MRI findings were age-appropriate.

**FIGURE 3:**
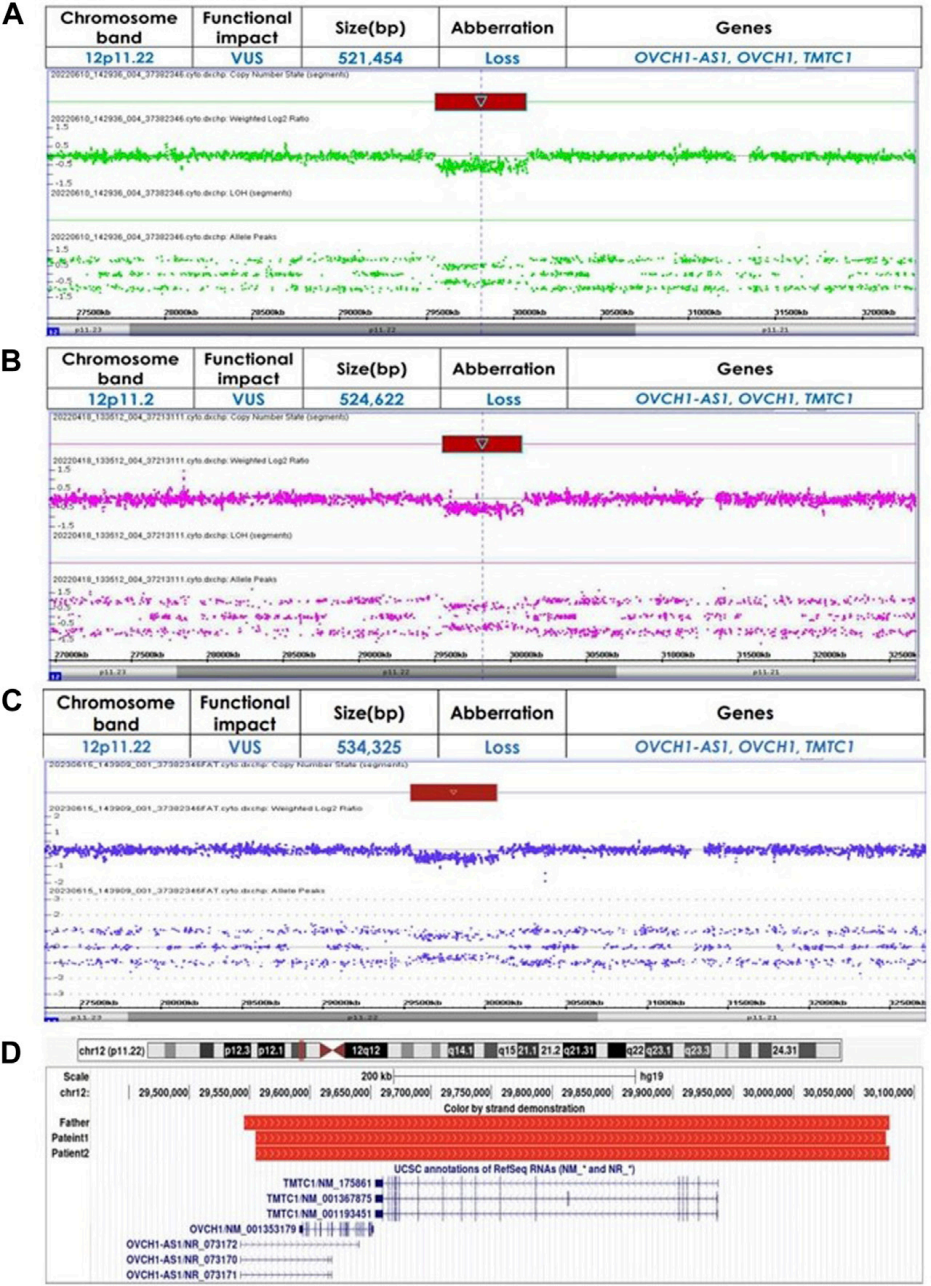
**(A)** Identified copy number variations using chromosomal microarray analysis in Patient 1 (chr12:29554804-30076258) **(B)** Identified copy number variations using chromosomal microarray analysis in Patient 2 (chr12:29554804-30079426) **(C)** Identified copy number variations using chromosomal microarray analysis in Father (chr12:29545101-30079426) **(D)** Chromosomal region 12p11.22 loss alignment, red bars represent CNVs identified in our patients and their father. Displayed at the bottom are the genes *OVCH1-AS1*, *OVCH1*, and *TMTC1*, as annotated in the UCSC Genome Browser [GRCh37/hg19 assembly].

Genetic investigations performed through CMA using an Affymetrix^®^ Cytoscan^®^ DX array (Affymetrix, USA) identified a 521,454 bp loss at 12p11.22 (chr12:29,554,804-30,076,258) including the *OVCH1-AS1*, *OVCH1*, and *TMTC1* genes, designated as a variant of unknown significance (VUS) (Figure 3A). There were no other CNVs detected. Identical CNVs found in the patient 1 were also observed in her younger sister (Patient 2) and her father but not in her mother (Figures 3B,C).

### 2.2 Patient 2

Patient 2, a 3-year-and 3-month-old female, first visited the outpatient clinic of the Catholic University of Korea, Eunpyeong St. Mary’s Hospital at 3 years of age with developmental delays, alongside her older sister (Patient 1). She was born as the second child to healthy nonconsanguineous Korean parents (Figure 1). The mother denied drinking alcohol, smoking or taking drugs during pregnancy. Patient 2 was delivered at 38 weeks of gestation via cesarean section, weighing 3.75 kg. Her assessment timeline and outcomes are detailed in Figure 2.

She achieved early language milestones, speaking her first word before 12 months and walking independently by 14 months, without notable motor development delays or seizure occurrences. However, similar to her sister, she exhibited developmental delays in both receptive and expressive language skills from 20 months, which were confirmed at her 24-month infant check-up; notably, she did not begin to put two words together until 36 months.

Following an early intervention with speech therapy starting at 26 months, she was diagnosed with BIF at 3 years old, with scores of FSIQ = 76, VC = 81, VS = 73, and WM = 83. Unlike her sister, Patient 2 did not exhibit conspicuous signs of ADHD according to the child behavior checklist (CBCL). Despite ongoing speech therapy initiated at 26 months, her language skills, both receptive and expressive, appeared to be below the 30-month level. Moreover, her expressive skills were significantly lower than her receptive skills. To date, she has been still able to speak only single words.

Furthermore, the IBS results indicated that her parents’ parenting and interactive behaviors were normal. Her mother described that the family have a good relationship and are close to each other. Clinically, she presented with no dysmorphic features, and brain MRI findings were normal for her age.

Genetic investigations performed through CMA identified a 524,622bp loss at 12p11.22 (chr12:29,554,804-30,079,426) including the *OVCH1-AS1*, *OVCH1*, and *TMTC1* genes, classified as a VUS (Figure 3B). There were no other CNVs in the genome. Identical CNVs found in the patient 2 were also observed in her older sister and her father (chr12:29,545,101-30,079,426, 534,325 bp loss) but not in her mother (Figures 3A,C). Figure 3D illustrates the overlapping regions of loss shared by the patients and their father.

## 3 Discussion

We report two individuals with 12p11.22 copy number loss involving the *OVCH1-AS1*, *OVCH1*, and *TMTC1* genes. The clinical features of our patients were mild ID and BIF, but neither presented brain structural abnormalities or any dimorphism, such as facial abnormalities and short stature. Genetic analysis identified a 521 kb loss (chr12:29,554,804-30,076,258) and a 525 kb loss (chr12:29,554,804-30,079,426) at 12p11.22, which was the only variant in both sisters. Moreover, identical variants were found in their father who exhibited probable BIF. Thus, the 12p11.22 variants may be associated with our patients’ cognitive impairments.

Multiple studies have described that chromosome 12 deletions are uncommon but are linked to ID, developmental delay, growth retardation and dysmorphic features (James et al., 2005; Adam et al., 2010; Lynch et al., 2011; Labonne et al., 2016; Deng et al., 2021). Unlike previously documented CNVs, the specific abnormalities identified in our patients were not found in established databases such as the DGV, dbVar, Gene2Phenotype, or DisGeNet. However, in the DECIPHER database (Firth et al., 2009), four cases with 12p deletions, including 12p11.22 loss was reported. Notably, one case, which shared a significant deletion overlap with our patients (DECIPHER 501071, chr12:27,895,481-30,065,521), was reported to be responsible for short stature. Among these, three cases were deemed to be likely pathogenic; one patient (DECIPHER 392769, chr12:21,155,799-34,703,759) was reported to have hydrocephalus, ID, and dysmorphic features such as short stature, broad and short neck, and bulbous nose (Boilly-Dartigalongue et al., 1985); one patient (DECIPHER 395971, chr12:19,855,799-33,155,799) reported hydrocephalus, mild ID, delayed speech and language development, and dysmorphic features such as short stature, short nose, and small hand (Nagai et al., 1995); and one patient (DECIPHER 400822, chr12:14,655,799-33,155,799) reported hydrocephalus, dysarthria, and dysmorphic features such as short stature and prominent nose. All three cases presented with hydrocephalus and dysmorphic features. Among them, two cases had ID and speaking or language problems.

When comparing the phenotypes described in these earlier cases with those of our patients, there are overlapping clinical symptom, such as problems with cognitive and language functioning. However, in the present study, we did not detect any dysmorphic features or hydrocephalus in our patients. Moreover, psychiatry and clinician assessments suggested that our patients’ deficits in language development were highly likely to result from ID. In fact, a previous study confirmed that ID is associated with a higher risk of speech and language delay in children, showing that 71.3% of children with ID have speech and language delay and 93.9% of children with moderate ID have speech and language problems (Memisevic and Hadzic, 2013). Despite these similarities, the gross chromosomal structural aberrations reported in the DECIPHER cases, which only partially overlap with those of our patients, encompass additional genes. This complicates the direct correlation between these CNVs and the observed phenotypes, underscoring the critical need for further research to elucidate their functions and implications.

The 12p11.22 region likely includes the genomic loci of candidate genes for cognitive impairment. This loss covering 3 genes at 12p11.22 probably harbors the genes responsible for the ID phenotypes shared between our patients and the cases from DECIPHER. *TMTC1* (transmembrane O-mannosyltransferase targeting cadherins) is one of four *TMTC* members, originally identified as an endoplasmic reticulum protein involved in calcium homeostasis. It has been reported to be involved in cognitive impairments related to schizophrenia (Mealer et al., 2020). Recently, *TMTC1* has been described as one of the candidate genes for neurodevelopmental disorder, particularly ID (Ben-Mahmoud et al., 2023). The expression of *TMTC1* mRNA is notably present in both adult and fetal brains, suggesting its relevance in these tissues (Ben-Mahmoud et al., 2023). Although *TMTC1* expression in the brain is not uniformly high, it shows moderate protein expression in the cerebral cortex and is cell type enhanced in inhibitory and excitatory neurons (Human Protein Atlas, proteinatlas.org) (Sjostedt et al., 2020), highlighting its potential role in the pathogenesis of diseases affecting these brain regions. Additionally, changes *TMTC1* expression, particularly in the striatum and cerebellar cortex during early life (Johnson et al., 2009), may contribute the intellectual disabilities observed (Balleine et al., 2007; Johnson et al., 2009), despite generally low levels in the cerebellar cortex and neocortex (Human Brain Transcriptome, hbatlas.org) (Johnson et al., 2009). *OVCH1* (ovochymase), another gene in the 12p11.22 region, encodes oocyte extracellular polyproteins (Mino and Sawada, 2016), which were reported to be involved in early-onset myasthenia gravis (Varade et al., 2018) and familial Meniere’s disease (Skarp et al., 2022), but its function in other cells remains unknown. Given the limited expression of these genes, further research is necessary to establish a clear correlation between the genes and phenotypes, including studies on gene expression regulation and the resulting phenotypic changes.

Interestingly, no variants other than the 12p11.22 loss including *OVCH1-AS1*, *OVCH1* and *TMTC1* were found in our patients. There were no structural abnormalities in brain development according to MRI or reported exogenous factors that could affect neurodevelopment, including delivery complications, exposure to drugs or alcohol during pregnancy, and parenting problems.

In addition, their father carried identical variants in the same region encompassing the same gene loss. Although he had never been diagnosed with ID, he reported experiencing delays in his cognitive and language development. He was told as a young child that his language development lagged behind that of peers and that his capacity for comprehension was similarly slower. This suggests that he might have been unaware that he presented with BIF.

According to the previously described findings, we propose that the 12p11.22 loss may be responsible for our patients’ mild ID and BIF. Additionally, the *OVCH1*, *OVCH1-AS1* and *TMTC1* variants identified in this study are the most likely disease-causing genes in the sisters. However, no links between ID and the *OVCH1* and *OVCH1-AS1* genes were found in the literature; thus, *TMTC1* seems to be a likely candidate gene for cognitive impairment. Hence, our findings can also be related to ID-associated variants, which would further support the diagnosis even if severity is mild.

For further study, since the father’s suspected BIF symptoms are based on self-report, an intelligence test would be helpful in clarifying his condition. Additionally, as he had struggled with BIF-related symptoms but is not currently experiencing severe difficulties, it would be helpful to follow up with the children to observe how their symptoms progress and what protective or risk factors they may have. In our study, Patient 2 was too young to complete the attention test, so there are limitations in clarifying the presence of ADHD symptoms in Patient 2. Although our study has some limitations, our findings may expand the limited knowledge on mild ID and BIF causative association in 12p11.22 loss and are highly helpful for identifying the genetic causes of ID even if symptoms are mild.

## Data Availability

The datasets for this article are not publicly available due to concerns regarding participant/patient anonymity. Requests to access the datasets should be directed to the corresponding author.
